# Perceptions about the Management of Patients with DM2 and COVID-19 in the Hospital Care Setting

**DOI:** 10.3390/jcm11154507

**Published:** 2022-08-02

**Authors:** Ricardo Gómez-Huelgas, Fernando Gómez-Peralta

**Affiliations:** 1 Servicio de Medicina Interna, Hospital Regional Universitario, Instituto de Investigación Biomédica de Málaga (IBIMA), Universidad de Málaga (UMA), 29010 Málaga, Spain; 2 Unidad de Endocrinología y Nutrición, Hospital General, Calle Luis Erik Clavería Neurólogo S/N, 40002 Segovia, Spain; fgomezp@saludcastillayleon.es

**Keywords:** diabetes mellitus, type 2, diabetes complications, coronavirus infections, hypoglycemic agents, hospitalization, ambulatory care, comorbidity

## Abstract

Background: COVID-19 entails a higher rate of complications in subjects with type 2 diabetes mellitus (T2DM). Likewise, COVID-19 infection can cause alterations in glucose metabolism that may lead to worse control. The aim of the study was to analyse the perceptions of a large group of Spanish physicians about the relationship between COVID-19 and T2DM, as well as the management, monitoring, and treatment of both diseases. Methods: A cross-sectional multicenter national project was conducted based on a survey which included opinion, attitude, and behavior (OAB) questions. Physicians specialised in internal medicine or endocrinology, whose usual clinical practices included the management of T2DM, responded to the survey between March and April 2021. Results: A total of 112 participants responded to the survey, from which 64.3% believed that COVID-19 entailed a higher risk of glycaemic decompensation irrespective of the presence of previously known T2DM. Obesity was considered a risk factor for poor control of T2DM by 57.7% and for a worse course of COVID-19 by 61.0%. Treatment intensification in not-on-target patients was considered by 57.1% in the presence of COVID-19 and by 73.2% in the absence of COVID-19. No participants considered the suspension of dipeptidyl peptidase 4 inhibitors (DPP-4i) in ambulatory patients, 85.7% declared that this therapeutic approach in hospitalized patients should be kept, and 88.4% supported the option of maintaining DPP-4i when corticosteroids were prescribed. Conclusion: The physicians involved in the management of T2DM and COVID-19 are aware of the bidirectional relationship between both conditions. However, the monitoring and therapeutic management of patients with T2DM who are infected by SARS-CoV-2 needs improvement through the following of the current recommendations and available evidence.

## 1. Introduction

Diabetes mellitus (DM) is a prevalent condition, affecting 9.3% of the worldwide population. Its prevalence has been constantly growing over the past 20 years, and it is expected to reach 10.9% of the population in 2045 [1,2]. In Spain, the prevalence of DM has been described as being even higher, reaching 13.8% of the inhabitants [3].

Patients with DM are at a higher risk of several infections, such as those of the lower respiratory tract, the urinary tract, and the skin and mucous, than patients without DM [4]. In the particular case of COVID-19, which has caused more than 11 million confirmed cases and 100,000 deaths in Spain [5], no greater risk of being infected by SARS-CoV-2 has yet been described in the population with DM. However, the SEMI-COVID-19 Registry found a higher prevalence of DM in patients hospitalized due to COVID-19 (19.4%) than in the general population [6]. Moreover, patients with DM have a higher rate of complications than subjects without DM when infected with SARS-CoV-2, such as severity, progression, hospital and intensive care unit (ICU) admissions, severe pneumonia, and mortality [7,8,9,10,11]. A plausible explanation for these outcomes is that chronic hyperglycaemia is associated with a chronic inflammatory state that can compromise the immune response [12]. Likewise, patients with SARS-CoV-2 and DM have increased levels of IL-6 and C-reactive protein (CRP), which can favour the systemic inflammatory response accompanying the typical acute respiratory distress syndrome in COVID-19 [10].

The relationship between DM and COVID-19 seems to be bidirectional as SARS-CoV-2 can cause alterations in glucose metabolism that may lead to the appearance of DM [13]. The underlying pathophysiological mechanism for this event might be the binding of SARS-CoV-2 to the ACE2 receptors in the pancreas (mainly in the islet cells), producing the dysfunction of β cells and acute hyperglycaemia [14]. In line with this, a greater risk of pancreatic injury has been observed among those patients with severe COVID-19 than those with a mild condition [14]. Moreover, the infection with COVID-19 can cause a wide range of sequelae, including DM, beyond the acute phase [15].

A proper blood glucose control seems to be important as hyperglycaemia, hypoglycaemia, and glycaemic variability can lead to worse outcomes in patients infected by SARS-CoV-2 [16,17,18,19,20,21]. In fact, glycaemia at admission due to COVID-19 is a powerful prognostic marker, not only in patients with DM but also in patients without diagnosed DM [21].

The global pandemic caused by the SARS-CoV-2 coronavirus infection has entailed a great impact on the routine care of patients with any chronic condition [22], as is the case with type 2 DM (T2DM). The requirements in terms of the clinical management of diabetes have probably changed after the COVID-19 pandemic: telemedicine, educational programs, strategies to ensure adherence, and glucose testing availability and affordability have become more necessary than ever [23].

In view of these new scenarios, it is crucial to optimise the management of patients with T2DM and COVID-19 in order to improve the prognosis and reduce the burden for health systems. The current study aimed to analyse the perception and experience of a large group of physicians involved in the management of these patients (internal medicine and endocrinology) on the relationship between COVID-19 and T2DM, their management, their monitoring, and their treatment of patients, whether hospitalized or not. Likewise, another objective was to identify potential differences between the current clinical practice and the recommendations of the scientific societies and expert panels and the best available evidence.

## 2. Materials and Methods

The present study is a cross-sectional multicentre national project based on a survey designed by a dedicated scientific committee, including several opinion, attitude, and behavior (OAB) questions (Appendix SA). This publication shows the results from a selection of 15 OAB questions related to (a) the relationship between T2DM and COVID-19; (b) the management of ambulatory patients with T2DM infected with COVID-19; (c) the management of patients with T2DM hospitalized due to COVID-19; and (d) the management of hyperglycaemia induced by corticosteroids (CS) in patients with COVID-19.

The participants were physicians specialised in internal medicine or endocrinology, whose usual practice included the management of T2DM. They were selected by means of a non-probabilistic directed sampling by conglomerates, according to proportional geographic and demographic distribution criteria. Data collection was out carried between the 15th of March and the 30th of April 2021 using an anonymous online questionnaire, completed by physicians in accordance with their usual practice. 

Regarding statistical methods and analysis, the sample was calculated according to a 95% confidence interval for a finite population proportion. The final sample size provided a precision level between ±7% and ±6.5% for a 95% confidence interval. With a reference population of 46,332,614 inhabitants and 217 hospitals with 100 beds or more, a sample of 108 physicians from different regions of Spain was estimated and was intended to be representative of the specialists in internal medicine and endocrinology in Spain. 

A descriptive statistical analysis was performed. The variables reported are qualitative and expressed as the values and percentages of multiple choice answers. Due to the exploratory nature of the study, no inferential statistics or regression analyses were performed. There were no missing data as the complete questionnaires were required. Statistical analysis was performed using the statistical program Stata v15.1 (StataCorp LLC, College Station, TX, USA).

## 3. Results

### 3.1. Participants

At the end of the field work, 112 invited participants had responded to all the survey items. We evaluated whether the final collected sample followed the estimated random sample, and we found that it was quite similar, although not exact, and supported the appropriateness of our sample. A proportion of 51.8% of them were 45 years old or younger; 67.9% were men. A total of 81.2% were specialists in internal medicine and 18.8% in endocrinology. The percentage of participants who used to work in hospitals with more than 300 beds was 59.8% (Table 1). 

### 3.2. COVID-19 and Type 2 Diabetes Mellitus Relationship

**Figure 1:**
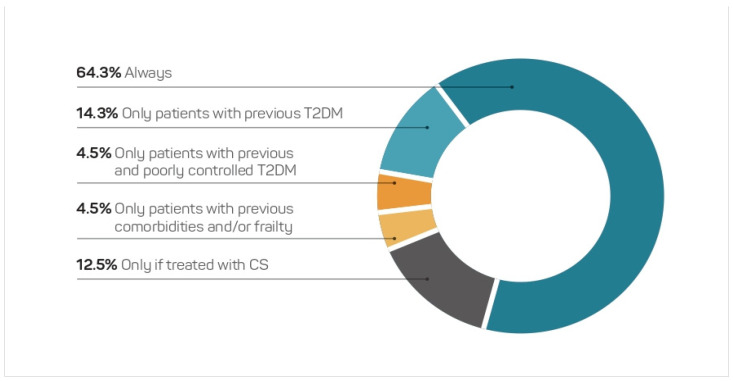
Opinion on whether patients with COVID-19 have a higher risk of glycaemic decompensation (*n* = 112). CS, Corticosteroids.

From the total pool of participants, 64.3% of the participants believed that COVID-19 always entailed a higher risk of glycaemic decompensation, while 14.3% considered it as such only in patients with known previous T2DM (Figure 1). Treatment with corticosteroids (CS), poor control of T2DM, and the presence of comorbidities were pointed to as factors for a higher risk of decompensation by 12.5%, 4.5%, and 4.5% of the participants, respectively. 

Likewise, 71.4% of the sample believed that T2DM was an independent risk factor for a bad prognosis of COVID-19, and 25.9% considered it as such only when T2DM was uncontrolled. The percentage of participants declaring that they had become stricter in terms of the objectives of T2DM control was 62.5%, while 37.5% answered that their clinical practice had not changed due to the appearance of COVID-19 (Figure S1).

The impact of several comorbidities on the control of T2DM and on the course of COVID-19 was inquired about among the participants; obesity was considered as a risk factor for poorer control of T2DM by 57.7% and for a worse course of COVID-19 by 61.0% of the participants. Frailty was considered as such by 13.5% and 10.5% and COPD by 10.6% and 11.4% of the sample, respectively (Table 2).

### 3.3. Ambulatory Patients with COVID-19 and Type 2 Diabetes Mellitus 

With regard to the recommendations about glycaemia monitoring in patients with T2DM and COVID-19 who did not require hospitalization, 73.2% of the participants declared an increase in the frequency of the controls, 25.0% stated that they had maintained it, and 0.9% that they had reduced it (Figure S2). The participants were also asked about the incorporation of new measures for the optimization of glycaemia control. The percentage of participants who responded that they had carried out treatment intensification in not-on-target patients with COVID-19 was 57.1%, and without COVID-19 was 73.2%. Likewise, 42.9% and 64.3% declared that they had insisted on diet and exercise recommendations in those patients with COVID-19 and without COVID-19, respectively (Table 3).

**Figure 2:**
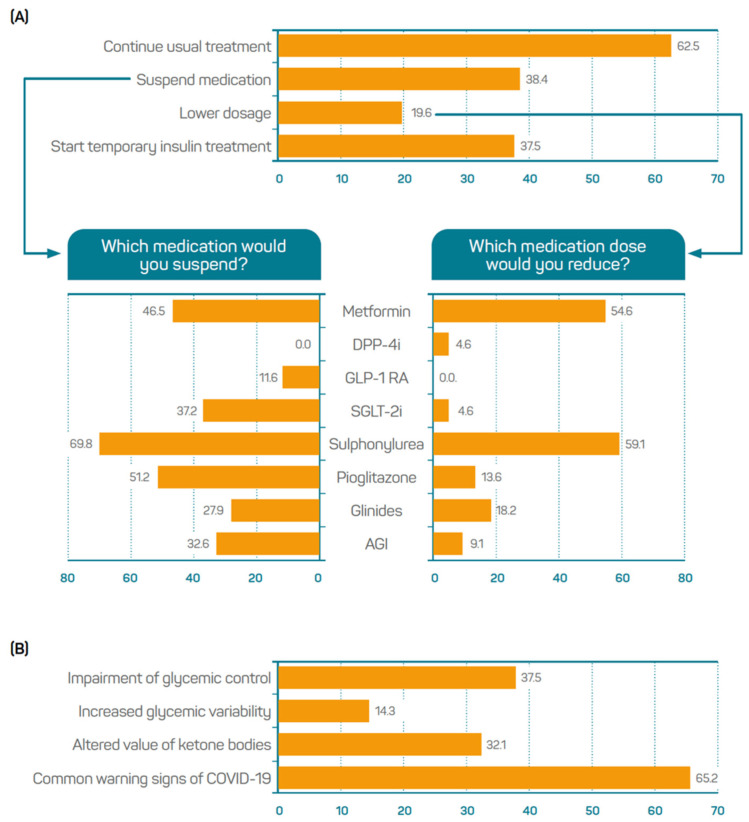
Opinion on management of ambulatory patients with T2DM and COVID-19: (**A**) therapeutic decisions made in these patients; (**B**) specific warning signs of T2DM to indicate hospital admission in these patients (*n* = 112). AGI: Alpha-glucosidase inhibitors; DPP-4i, Dipeptidyl peptidase 4 inhibitors; GLP-1 RA, Glucagon-like peptide 1 receptor agonists; SGLT2i, Sodium-glucose transport protein 2 inhibitors.

The participants gave their opinion on the therapeutic management of ambulatory patients with T2DM and COVID-19, and 62.5% of the participants indicated that they had maintained the usual treatment. From the 38.4% of participants who declared that they had suspended medication sometimes, 69.8% pointed at sulphonylureas, 51.2% at pioglitazone, and 46.5% at metformin. No participants considered the suspension of dipeptidyl peptidase 4 inhibitors (DPP-4i). From the 19.6% of participants who declared that they had reduced medication dosing sometimes, 59.1% pointed at sulphonylureas and 54.6% at metformin. iDPP4 and sodium-glucose transport protein 2 inhibitors (SGLT2i) were selected by 4.6% and glucagon-like peptide 1 receptor agonists (GLP-1 RA) by none of the participants (Figure 2A). The participants were asked to indicate which warning signs should be considered as an indication for hospital admission. The common warning signs of COVID-19, such as fever, cough, tiredness, and loss of taste or smell were selected by 65.2% of the participants. Impaired glycaemic control and altered ketone bodies values were indicated by 37.5% and 32.1% of the sample, respectively (Figure 2B).

### 3.4. Patients Hospitalized Due to COVID-19 with Type 2 Diabetes Mellitus

The participants gave answers regarding the patients for whom they thought hyperglycaemia at admission meant a worse prognosis: 68% of them agreed that it was worse in patients with both known and unknown T2DM; 23% stated that it was worse only in patients with known T2DM; and 5% said that it was worse only in patients with unknown T2DM (Figure S3).

**Figure 3:**
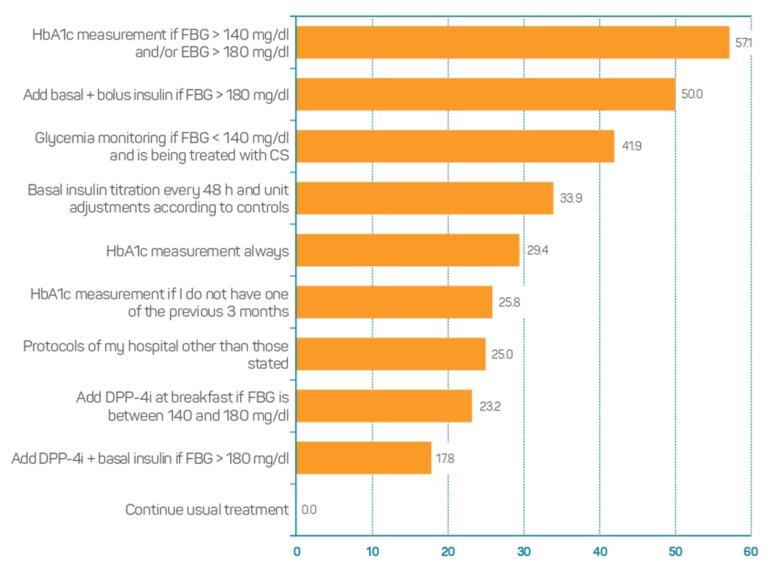
Opinion on therapeutic management of patients with unknown T2DM admitted to hospital due to COVID-19, who presented with hyperglycemia and did not require corticosteroids (*n* = 112. CS, Corticosteroids; DPP-4i, Dipeptidyl peptidase 4 inhibitors; EBG, evening blood glucose; FBG, fasting blood glucose; HbA1c, Glycated haemoglobin.

In patients with unknown T2DM admitted to hospital due to COVID-19, who presented with hyperglycemia and did not require CS, 57.1% of the participants answered that they had requested a determination of HbA1C if the basal blood glucose was >140 mg/dL and/or the evening blood glucose was >180 mg/dL. Moreover, 50.0% of them coincided in adding DPP4i and basal insulin if the basal glycaemia exceeded 180 mg/dL (Figure 3).

**Figure 4:**
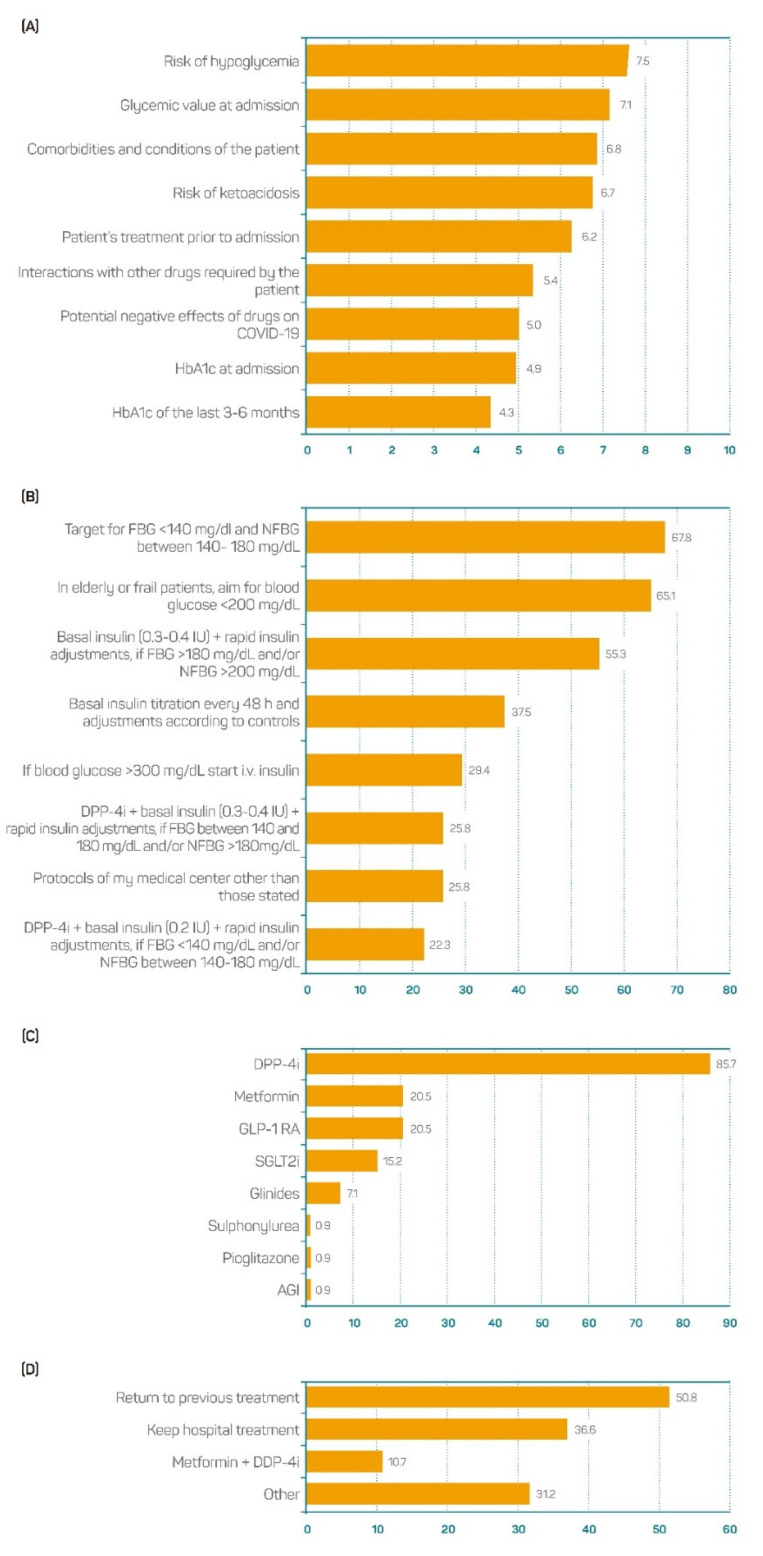
Opinion on clinical practice in patients with known T2DM admitted to hospital due to COVID-19: (**A**) factors to consider when choosing a glucose-lowering treatment; (**B**) criteria to consider when treating hyperglycemia; (**C**) pharmacological class maintained during hospitalization; (**D**) glucose-lowering approach taken at discharge (*n* = 112). AGI: Alpha-glucosidase inhibitors; DPP-4i, Dipeptidyl peptidase 4 inhibitors; FBG, fasting blood glucose; GLP-1 RA, Glucagon-like peptide 1 receptor agonists; HbA1c, Glycated haemoglobin; IU, International units; NFBG, non-fasting blood glucose: SGLT2i, Sodium-glucose transport protein 2 inhibitors.

In the patients with known T2DM admitted to hospital due to COVID-19, the risk of hypoglycemia, the value of glycemia at admission, the presence of comorbidities and conditions, and the risk of ketoacidosis were the four most important factors to consider when a glucose-lowering treatment was prescribed (Figure 4A). 

Considering the patients with T2DM hospitalized due to COVID-19, 67.8% of the participants were in favour of setting a fasting glucose target < 140 mg/dL and 140–180 mg/dL during the rest of the day. The percentage of participants in favour of considering milder goals (<200 mg/dL) in elderly or frail patients was 65.1%. The percentage of participants declaring that they had prescribed basal insulin and rapid insulin corrections when required to patients with basal glycaemia > 180 mg/dL and/or non-basal glycaemia > 200 mg/dL was 55.3% (Figure 4B). 

From the total sample, 85.7% chose to keep the current glucose-lowering treatment when this was DPP4i, while other therapeutic options, such as metformin or GLP-1 Ras, were only considered by 20.5%. The maintenance of other options, such as sulfonylurea, pioglitazone, or an alpha-glucosidase inhibitor (AGI), was supported by 0.9% of the participants (Figure 4C). When the participants were asked about what glucose-lowering approach they considered at discharge, 50.8% declared that they had returned to the previous treatment, 36.6% coincided with keeping the treatment prescribed at the hospital, and 10.7% stated that they had prescribed metformin plus DPP-4i (Figure 4D).

### 3.5. Corticosteroids-Induced Hyperglycaemia in Patients with COVID-19

**Figure 5:**
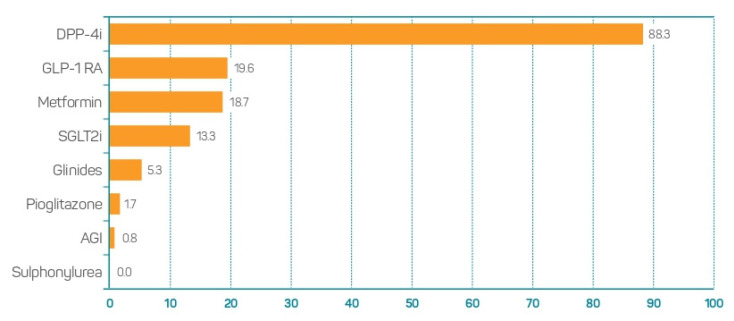
Opinion on pharmacological classes to be maintained in patients with known T2DM admitted to hospital due to COVID-19 and requiring corticosteroids. AGI: Alpha-glucosidase inhibitors; DPP-4i, Dipeptidyl peptidase 4 inhibitors; GLP-1 RA, Glucagon-like peptide 1 receptor agonists; SGLT2i, Sodium-glucose transport protein 2 inhibitors.

In those patients with previously known T2DM who were admitted to hospital due to COVID-19 and required treatment with CS, 88.4% of the participants supported the option of maintaining the treatment with DPP-4i; the maintenance of metformin was supported by 18.8% and of GLP-1 RA by 19.6%. None of the participants supported the maintenance of sulphonylureas (Figure 5).

The criteria for the treatment of CS-induced hyperglycaemia in patients with COVID-19 were also inquired about. In the patients with unknown T2DM at the time of hyperglycaemia onset, 76.8% considered it critical to control glycaemia within the days after starting the treatment with CS, and 61.6% supported objectives of <140 mg/dL for basal glycaemia and of <200 mg/dL for postprandial glycaemia. These percentages were 86.6% and 57.1% when considering patients with a previous diagnosis of T2DM (Figure S4).

## 4. Discussion

The current publication provides an overview of the perception and the current clinical practice in patients with T2DM who are infected with SARS-CoV-2 in the Spanish setting. In general terms, the majority of the participants believed that COVID-19 itself entailed a higher risk of T2DM decompensation and T2DM new onset, as has been supported by recent evidence [15]. As previously described, both the SARS-CoV-2 infection and the therapy administered at admission, frequently including CS, increase the risk of glycaemic decompensation [21,24]. A plausible explanation for this is the presence and replication of the virus in the pancreatic islets [25,26] and the inflammation process generated by COVID-19 [27], which could lead to insulin resistance. Some authors have hypothesized that new-onset diabetes in patients with COVID-19 has a multifactorial nature and could stem from factors that induce autoimmunity, β-cell stress, insulin resistance, and local hypoxia and from inflammation that damages β-cells [28]. At the same time, hyperglycemia is associated with the need for mechanical ventilation and ICU admission and with mortality [21] in patients with COVID-19; so, it is important to reduce its risk. Despite these data, and although hyperglycaemia is a factor of bad prognosis in all patients with COVID-19, particularly in those with no diagnosis of T2DM [21,29], nearly 15% of the sample believed that it only affected those patients with previously diagnosed T2DM. A similar percentage of participants believed that the risk of hyperglycaemia was only increased for those patients treated with CS. 

DM is one of the most prevalent comorbidities in patients hospitalized due to COVID-19 in Spain, being present in 19.4% of patients with the infection [6]. DM has been described as a risk factor for a bad prognosis of COVID-19 [20,30]. In this study, 71.4% of the sample considered T2DM as an independent risk factor, and the rest took into consideration other conditioning factors, such as poor control, comorbidities, or frailty. The percentage of participants who declared that they had not modified their usual clinical practice in light of this fact was 37.5%, probably meaning a problem of therapeutic inertia when it comes to managing patients with T2DM infected by SARS-CoV-2. 

Obesity is a highly prevalent condition in patients with severe manifestations of COVID-19 [31]. In patients with T2DM, obesity also increases the risk of poor glycemic control, and it is probably the comorbidity with the greatest impact on COVID-19 prognosis [32]. However, around 40% of the participants did not identify it as a risk factor for poorer control of T2DM and a worse course of COVID-19 in patients with both diseases. Also remarkable was the low number of participants rating comorbidities such as hypertension or heart disease as reasons for a worse course of COVID-19 in patients with T2DM, even though they have been described as such in populations with T2DM, regardless of COVID-19 disease [33].

The COVID-19 pandemic and the resulting lockdowns and behavioral changes could have exerted an impact on the glycaemic control of patients with T2DM [34], although the data from a large database in the USA showed no differences between HbA1c levels between the pandemic period and the previous year [35]. In the current work, 73.2% of the participants recommended increasing the frequency of glycaemia controls in SARS-CoV-2-infected non-hospitalized patients. With regard to the glucose-lowering treatment of patients who are affected by COVID-19, a large retrospective study conducted in Germany showed a negative impact of the pandemic on T2DM patients’ care: the intensification of the treatment with any therapeutic option was reduced. The number of uncontrolled patients who underwent at least one therapeutic regimen change was reduced too [36]. From the measures for the optimization of glycemic control, 73.2% of the participants chose the intensification of patients without COVID-19 who were not on target, and 57.1% chose the patients with COVID-19. Thus, a relevant part of the sample did not agree with this measure, although it is recommended in the main international guidelines [37] and, in the case of patients with COVID-19, it helps to avoid severe manifestations. Moreover, higher levels of HbA1c are associated with systemic inflammation, hypercoagulability, and bad prognosis of COVID-19 [17], and hyperglycemia has also been shown to have a clear negative impact on mortality in hospitalized patients with COVID-19 [21].

The participants also gave their opinion on the use of different glucose-lowering therapeutic options in patients with T2DM and COVID-19. In this regard, GLP1 RA and SGLT2i could be an inappropriate option as they can induce overexpression of angiotensin-converting enzyme 2 (ACE2) [38], the receptor by which SARS-CoV-2 attacks pneumocytes [39]. Moreover, the discontinuation of SGLT2i is recommended at hospital admission, as it can increase the risk of diabetic ketoacidosis, urinary and genital infections, and volume depletion [40]. GLP1 RA should be used with caution, as long as dehydration does not occur, and by always encouraging adequate fluid intake and regular meals [41]. Its discontinuation should be considered in patients with long-term disease and intestinal symptoms [42]. Sulfonylureas increase the risk of hypoglycemia and are discouraged in hospitalized patients with severe disease. The use of metformin possesses a certain risk of lactic acidosis in patients with hypoxia and acute disease [43], and it is consequently contraindicated in patients with respiratory problems and hypoxia. Some authors advise a careful monitoring of kidney disease and also the withdrawal of metformin in dehydrated patients as there is a risk of lactic acidosis [41], and some others state its contraindication in patients hospitalized due to COVID-19 [40]. Different meta-analyses have reported a negative association with DPP-4 inhibitor use and a risk of mortality. DPP-4i may represent a good option for preventing and reducing the complications of SARS-CoV-2 infection [12,44,45]. However, given the observational nature of the available studies, the possible benefits of using any antidiabetic agent should be addressed [12,44]. In line with these previous findings, the proportion of physicians who declared that they had suspended or reduced the treatment with DPP-4i in ambulatory patients was imperceptible, and this therapeutic option was the most valued when the participants were asked about which glucose-lowering drug would be maintained in the case of hospitalization, even in patients requiring CS therapy.

The use of CS in patients with pre-existing T2DM results in a worsening of glycemic control [46], and it is the main cause of hyperglycemic decompensation in hospitalized patients. For this reason, glycaemia needs to be narrowly monitored and treated accordingly in all patients under CS treatment [46,47]. CS is a frequently prescribed medication in patients with COVID-19 because of its effect on hyperinflammation and the potential reduction in mortality [48]. Even so, the percentage of participants who did not agree with controlling glycaemia during the days after introducing CS was 23.2% when it came to patients with known T2DM, and 13.4% with unknown T2DM. Consequently, an important proportion of physicians managing patients with T2DM and COVID-19 might not be following the current recommendations and increasing the risk of hyperglycemia in these patients.

This study entails certain limitations related to its qualitative nature and its design. First, there was not a randomized selection, and this sample might not be representative of the whole population of Spanish physicians specialised in internal medicine or endocrinology. Consequently, the generalizations are limited; another sample may reach different conclusions. However, we used the published data by the Spanish Ministry of Health of 2008 to know the approximate distribution of internists and endocrinologists and the female percentage for both specialties in Spanish hospitals, and by indirect comparison, our sample was not very far from the published data. 

Second, the number of specialists in internal medicine was greater than the number of specialists in endocrinology. Once again, the purpose was not to compare results among specialties, but to obtain the global opinion of physicians. Third, the survey was designed with pre-defined answers, which could have made it difficult to contribute with personal ideas or clarifications. In this regard, the inclusion of more answers or an open field would have supposed a higher risk of an excessive dispersion of answers. To counteract this limitation, the survey was designed by a scientific committee including specialists in both internal medicine and endocrinology, thus ensuring the inclusion of those answers considered to be more relevant. Last, but not least, the interpretation of the statements or options of response could have been variable among the participants, but in any case, the questionnaire was reviewed by experts belonging to both endocrinology and internal medicine specialties.

## 5. Conclusions

The appearance of COVID-19 has impacted on the clinical outcomes of patients with T2DM. Thus, clinical management needs to be adapted to the new reality. Physicians involved in this study seem to be aware of the bidirectional relationship between both T2DM and the COVID-19/coronavirus infection. However, there is still room for improvement in terms of the monitoring and therapeutic management of patients with T2DM who are infected by SARS-CoV-2. It is important to put emphasis on spreading the available evidence, following current recommendations, and favouring practices that ensure an adequate metabolic control and that minimize the risk of complications and the hospitalization of these patients. Although a randomized sample was desirable, the data obtained from this large panel group of Spanish physicians concerning T2DM and COVID-19 management, and the way in which the results are consistent with those suggested by the guidelines or known evidence, can contribute to the identification of key issues and trends to explore in further studies in order to identify strategies aimed at optimizing the clinical practice.

## Figures and Tables

**Table 1:** Characteristics of participants (*n* = 112).

		*n* (%)
**Age**	30–45	58 (51.8)
	46–55	27 (24.1)
	56–65	26 (23.2)
	>65	1 (0.9)
**Gender**	Women	36 (32.1)
	Men	76 (67.9)
**Medical specialty**	Internal medicine	91 (81.2)
	Endocrinology	21 (18.8)
**Work centre**	<100 beds	13 (11.6)
	100–200 beds	18 (16.1)
	201–300 beds	14 (12.5)
	>300 beds	67 (59.8)
**Participation in COVID-19/DM data analysis initiatives**	None	95 (84.8)
	Collaborative Open-Access Virtual Database for COVID-19 in Diabetes	2 (1.8)
	Others	15 (13.4)
**Training/update in the management of COVID-19/DM**	Hospital protocols	71 (63.4)
	Clinical sessions	65 (58.0)
	Bibliography	82 (73.2)
	Webinars	70 (62.5)
	Pharmaceutical company initiatives	45 (40.2)
	Courses	48 (42.9)
	Task forces in scientific societies	75 (67.0)

**Table 2:** Opinion on the impact of comorbidities on T2DM control or on the course of COVID-19 in patients with both diseases.

Comorbidity	% of Responses	
	Poorer Control of T2DM (*n* = 104)	Worse Course of COVID-19 (*n* = 105)
**Obesity**	57.7	61.0
**Frailty**	13.5	10.5
**COPD**	10.6	11.4
**Renal insufficiency**	7.7	4.8
**Heart disease**	6.7	8.6
**Hypertension**	2.9	2.9
**Oncohematological disease**	1.0	1.0

**Table 3:** Opinion on measures incorporated in clinical practice for the optimization of glycaemic control in ambulatory patients with T2DM (*n* = 112).

Measures	% of Agreement	
	Patients with COVID-19	Patients without COVID-19
**Carry out treatment intensification if the patient is not on target**	57.1	73.2
**Insist on recommendations about diet and exercise**	42.9	64.3
**Frequent self-monitoring of glucose**	42.0	27.7
**Stricter control targets if well tolerated**	28.6	33.9
**Monitor glycemic variability in controls**	25.9	25.9
**More frequent HbA1c controls to confirm degree of control**	17.7	25.0
**Continuous glucose monitoring systems**	4.5	5.4

## Data Availability

The majority of the data are contained within the article or supplementary material. The datasets generated during and/or analysed during the current study are available from the corresponding author upon reasonable request.

## Supplementary Materials

The following supporting information can be downloaded at: https://www.mdpi.com/article/10.3390/jcm11154507/s1, Figure S1: opinion on (a) the consideration of T2DM as an independent risk factor of COVID-19 bad prognosis and (b) its impact in clinical practice (*n* = 112), Figure S2: opinion on recommendations of blood glucose monitoring of ambulatory patients with DM2 and COVID-19 (*n* = 112), Figure S3: opinion on which hospitalized patients have a worse prognosis when hyperglycaemia at admission is present (*n* = 112), Figure S4: opinion on criteria for the treatment of corticosteroid-induced hyperglycaemia in patients with COVID-19 (a) with unknown T2DM and (b) with known T2DM (*n* = 112).
