# Supplementary material for: Perceptions about the Management of Patients with DM2 and COVID-19 in the Hospital Care Setting
Source: J Clin Med. 2022 Aug 2;11(15):4507. doi: 10.3390/jcm11154507 (PMC9369585; doi:10.3390/jcm11154507)
Supplement: Supplementary file 1 [file jcm-11-04507-s001.zip › jcm-1816269-supplementary.pdf]

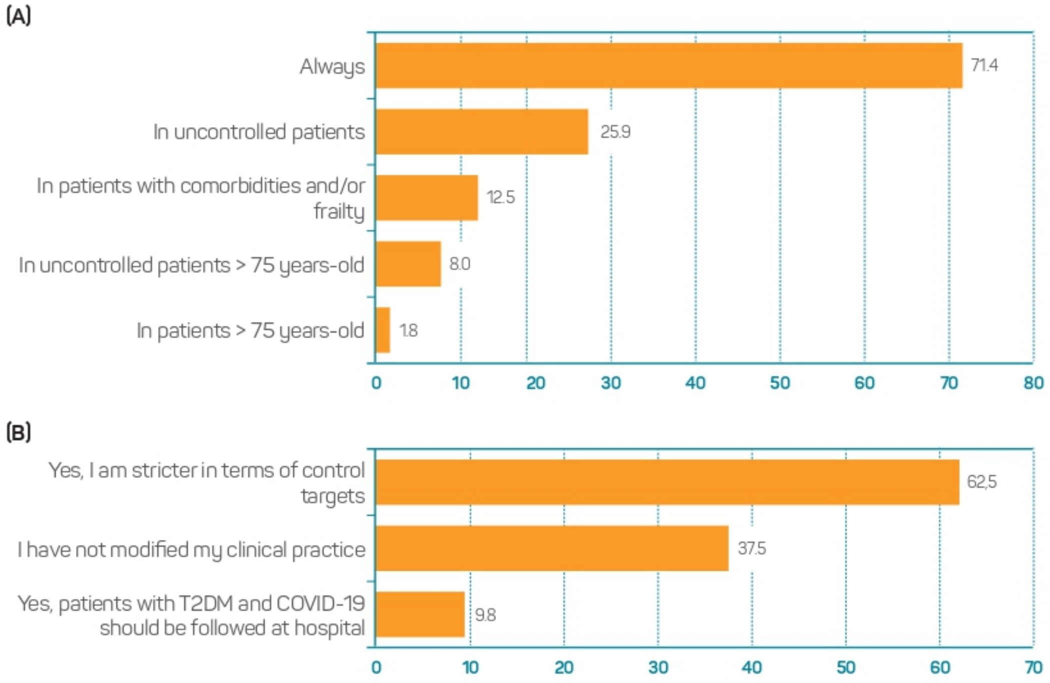

Figure S1. Opinion about (**A**) the consideration of T2DM as an independent risk factor of COVID-19 bad prognosis and (**B**) its impact in clinical practice (n = 112).

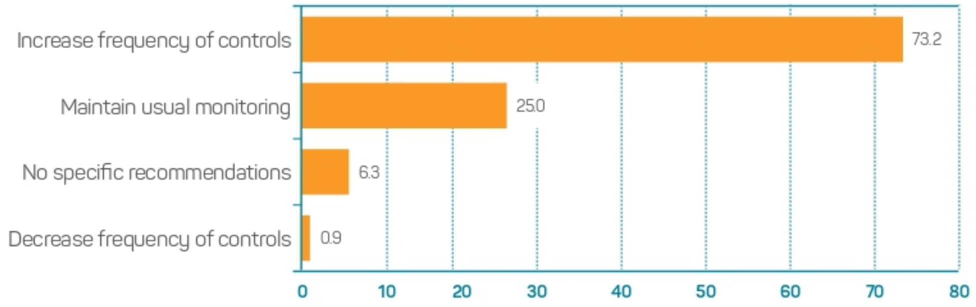

Figure S2. Opinion about recommendations of blood glucose monitoring of ambulatory patients with DM2 and COVID-19 (n = 112).

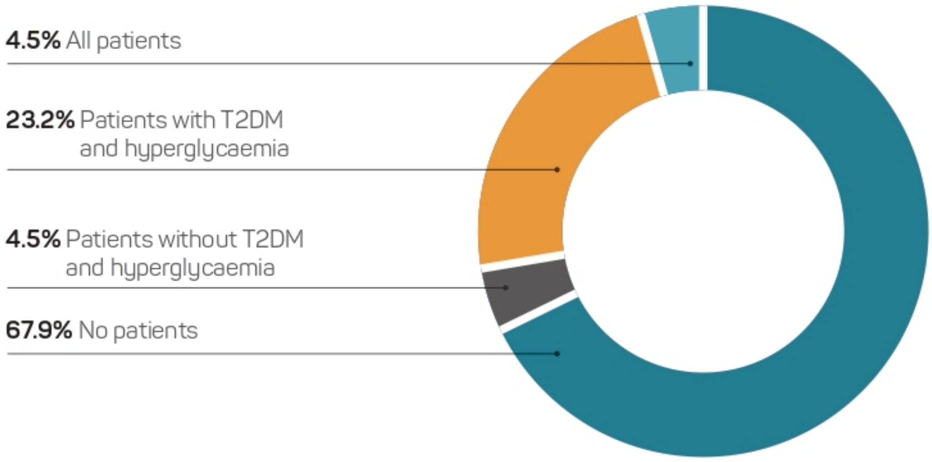

Figure S3. Opinion about what hospitalized patients have a worse prognosis when hyperglycaemia at admission is present (n = 112).

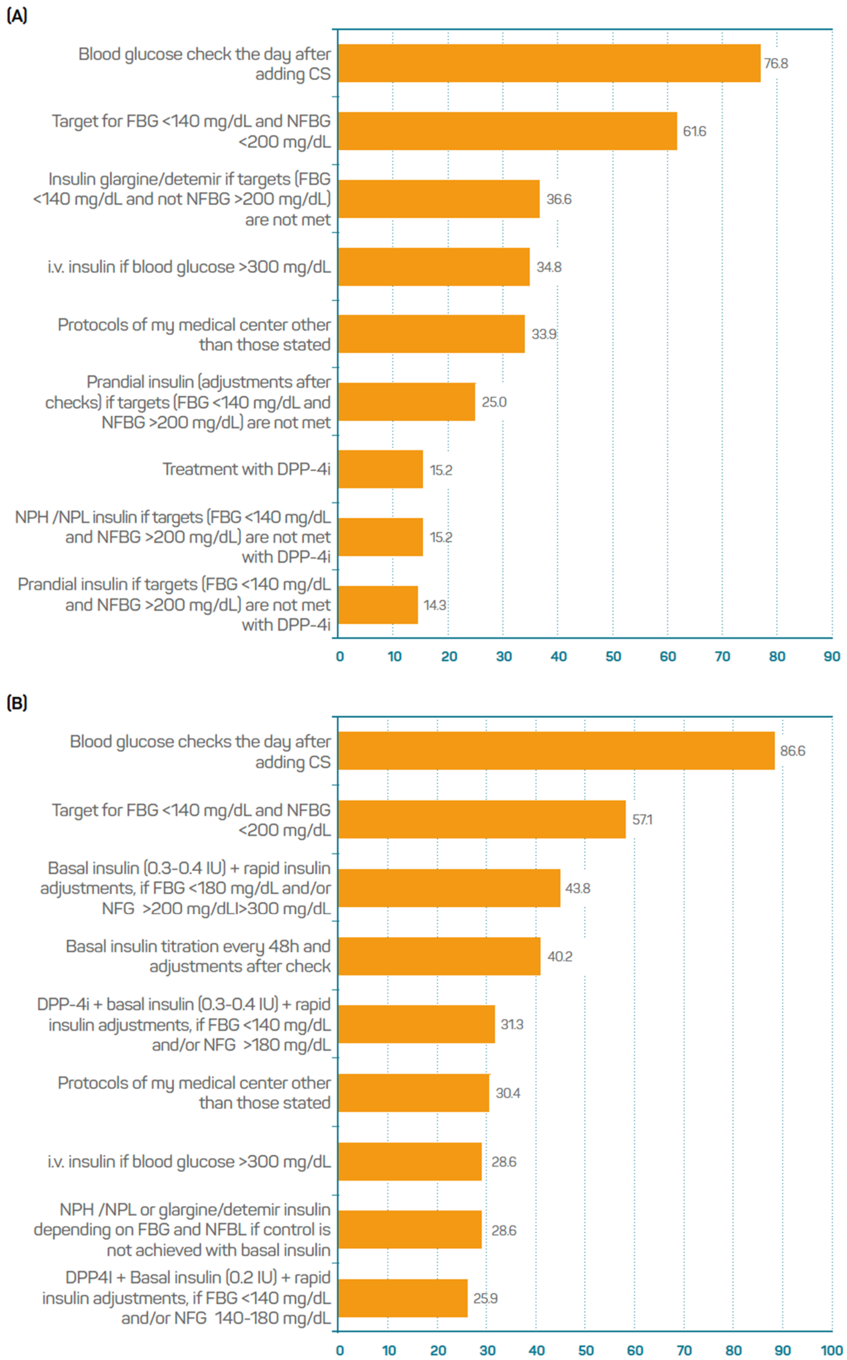

Figure S4. Opinion about criteria for the treatment of corticosteroids-induced hyperglycaemia in patients with COVID-19 (**A**) with unknown T2DM and (**B**) with known T2DM (n = 112). CS, Corticosteroids; DPP-4i, Dipeptidyl peptidase 4 inhibitors; EBG, evening blood glucose; FBG, fasting blood glucose; NFBG, non-fasting blood glucose.

Appendix SA. Survey Questionnaire

PERFIL DEL MÉDICO

I. **Especialidad:**

- ☐ Medicina Interna
- ☐ Endocrinología

II. **Edad: _ _ (años)**

III. **Sexo:**

- ☐ Mujer
- ☐ Hombre

IV. **Comunidad autónoma (lugar de trabajo):**

- ☐ Andalucía
- ☐ Aragón
- ☐ Asturias (Principado de)
- ☐ Balears (Illes)
- ☐ Canarias
- ☐ Cantabria
- ☐ Castilla-La Mancha
- ☐ Catalunya
- ☐ Castilla y León
- ☐ Comunitat Valenciana
- ☐ Extremadura
- ☐ Galicia
- ☐ Madrid (Comunidad de)
- ☐ Murcia (Región de)
- ☐ Navarra (Comunidad Foral de)
- ☐ País Vasco
- ☐ Rioja (La)
- ☐ Ceuta (Ciudad de)
- ☐ Melilla (Ciudad de)

V. **¿Cómo se define el hospital en el que usted trabaja la mayor parte del tiempo?**

- ☐ <100 camas
- ☐ 100–200 camas
- ☐ 201–300 camas
- ☐ >300 camas

VI. **¿Cómo se forma/actualiza habitualmente en el manejo de las distintas situaciones clínicas en el contexto DM2-COVlD-19?** Puede señalar más de una.

- ☐ Protocolos propios de mi entorno de trabajo
- ☐ Sesiones clínicas
- ☐ Lectura de bibliografía
- ☐ «Webinars»
- ☐ Iniciativas lideradas por la industria farmacéutica
- ☐ Cursos de formación
- ☐ Documentos de grupos de trabajo o sociedades

VII. **¿Ha participado en alguna iniciativa colaborativa para el análisis de datos COVID y DM?**

- ☐ No
- ☐ Sí, he participado en Collaborative Open-Access Virtual Database forCOVID-19 In Diabetes: https://www.covidindiabetes.org (n).
- ☐ Sí, he participado en otras iniciativas distintas a Collaborative Open-Access Virtual Database for COVID-19 In Diabetes

VIII. **¿Ha tratado directamente pacientes con COVID-19 hospitalizados?**

- ☐ Sí
- ☐ No

PREGUNTAS CRD

En relación con los siguientes aspectos, indique la opción que más se adecua con su opinión y experiencia considerando la situación de práctica habitual durante la pandemia de la COVID-19 Si para alguna pregunta se permite señalar más de una respuesta, se indicará expresamente en el enunciado.

BLOQUE 1. DM2 y COVID. Generalidades y recursos

1. **¿Es la DM2 un factor de riesgo independiente para el mal pronóstico de COVID-19? Puede señalar más de una opción.**
   - ☐ Sí, siempre
   - ☐ Sí, pero solo en pacientes mayores de 75 años C] Sí, si está mal controlada
   - ☐ Si está mal controlada en mayores de 75 años
   - ☐ Solamente cuando se asocia a otras comorbilidades g/o fragilidad
2. **¿Ha impactado en su práctica habitual la consideración de la DM2 como factor de riesgo independiente para el mal pronóstico de la COVID-19? Puede señalar más de una opción.**
   - ☐ Desde el inicio de la pandemia, en los pacientes con diabetes soy más exigente en alcanzar objetivos de buen control y realizo los ajustes necesarios para conseguirlo
   - ☐ No he modificado la práctica habitual sobre objetivos de control a raíz de la pandemia de la COVID-19
   - ☐ Considero que todos los pacientes con DM2 e infección por SARS-CoV-2 deberían ser seguidos a nivel hospitalario
3. **¿Ha observado un mayor riesgo de desarrollo de COVID-19 en los pacientes con DM2 y otras comorbilidades?**
   - ☐ No
   - ☐ Sí
   - ☐ Sí, pero solamente si la DM2 no está bien controlada.
4. **En caso de haber respondido "sí" a alguna de las opciones de la pregunta 3, indique con cuál de las siguientes comorbilidades ha observado mayor riesgo de desarrollo de COVID-19 en pacientes con DM2/DM2 mal controlada.**

☐ Obesidad	☐ Insuficiencia renal
☐ Dislipemia	☐ Fragilidad
☐ Hipertensión	☐ EPOC
☐ Enfermedad cardiovascular	☐ Enfermedad oncohematológica

5. **En su experiencia, en un paciente con COVID ¿considera que la DM2 se controla peor si el paciente presenta otras comorbilidades?**
   - ☐ No
   - ☐ Sí
6. **En caso de haber respondido "sí" en la pregunta 5, indique cuál de las siguientes comorbilidades relaciona con un peor control de la DM2 en pacientes con COVID-19.**
   - ☐ Obesidad
   - ☐ Insuficiencia renal
   - ☐ Dislipemia

- ☐ Fragilidad
- ☐ Hipertensión
- ☐ EPOC
- ☐ Enfermedad cardiovascular
- ☐ Enfermedad oncohematológica

7. **¿Ha observado peor evolución de la COVID-19 en pacientes con DM2 y otras comorbilidades?**
   - ☐ No
   - ☐ Sí

8. **En caso de haber respondido "sí" en la pregunta 7, indique cuál de las siguientes comorbilidades relaciona con una peor evolución de la COVID-19 en pacientes con DM2.**

☐ Obesidad	☐ Insuficiencia renal
☐ Dislipemia	☐ Fragilidad
☐ Hipertensión	☐ EPOC
☐ Enfermedad cardiovascular	☐ Enfermedad oncohematológica

BLOQUE 2. Paciente ambulatorio

9. **¿Existe en su centro de trabajo/área sanitaria algún sistema que permita la monitorización en remoto del paciente con DM2 y COVID en domicilio?**
   - ☐ No
   - ☐ Lo desconozco
   - ☐ Sí (indicar cuál/cuáles de las siguientes opciones):
   - ☐ Teléfono
   - ☐ Correo electrónico
   - ☐ Plataforma específica de videollamada
   - ☐ Plataformas externas
   - ☐ Programas de descarga de datos de monitorización continua de glucemia

10. **Indique lo que mejor se ajuste a su realidad en relación con el desarrollo de la e-consulta (sin considerar como tal teléfono o correo electrónico) entre atención primaria y especialidades hospitalarias en su área sanitaria:**
    - ☐ Ya estaba desarrollado antes de la pandemia de la COVID-19
    - ☐ La COVID-19 ha catalizado su desarrollo
    - ☐ No tenemos disponible por ahora la e-consulta entre especialidades

11. **En relación con la optimización del control glucémico en el paciente ambulatorio con DM2 sin infección por SARS CoV-2, ¿ha cambiado su actitud habitual incorporando alguna de las siguientes medidas?** Puede señalar más de una opción.
    - ☐ HbA1c con mayor frecuencia para confirmar grado de control
    - ☐ Fijo objetivos de control más estrictos si son bien tolerados
    - ☐ Indico automedidas de la glucosa más frecuentes
    - ☐ Indico sistemas de monitorización continua de la glucosa
    - ☐ Vigilo la variabilidad glucémica en los controles
    - ☐ Realizo intensificación del tratamiento si el paciente no está en objetivos
    - ☐ Insisto en evaluación y recomendaciones sobre dieta y ejercicio

12. **En relación con la optimización del control glucémico en el paciente ambulatorio con DM2 y COVID-19, ¿ha cambiado su actitud habitual incorporando alguna de las siguientes medidas?** Puede señalar más de una opción**.**
    - ☐ HbA1c con mayor frecuencia para confirmar grado de control
    - ☐ Fijo objetivos de control más estrictos si son bien tolerados
    - ☐ Indico automedidas de la glucosa más frecuentes

- [ ] Indico sistemas de monitorización continua de la glucosa
- [ ] Vigilo la variabilidad glucémica en los controles
- [ ] Realizo intensificación del tratamiento si el paciente no está en objetivos
- [ ] Insisto en evaluación y recomendaciones sobre dieta y ejercicio
- [ ] No ha cambiado mi manejo

13. **En el paciente con DM2 que contrae COVID-19 y no precisa ingreso hospitalario, ¿qué recomendaciones ofrece habitualmente sobre monitorización de la glucemia?** Puede señalar más de una opción.

- [ ] Indico mantener la monitorización habitual
- [ ] Aconsejo incrementar la frecuencia, al menos 2–4 controles al día, o bien si hay síntomas compatibles con descompensación
- [ ] Indico hacer menos controles porque es normal que la glucemia esté descompensada por la infección
- [ ] No realizo ninguna recomendación específica

14. **En un paciente con DM2 ya conocido que contrae COVID-19 y no precisa ingreso hospitalario, ¿con cuáles de las siguientes decisiones sobre el tratamiento hipoglucemiante está de acuerdo?** Puede señalar más de una opción.

- [ ] Indico mantener el tratamiento habitual
- [ ] Aconsejo suspender (puede señalar más de una)
- [ ] Metformina
- [ ] iDPP4
- [ ] arGLP1
- [ ] iSCLT2
- [ ] Sulfonilureas
- [ ] Pioglitazona
- [ ] Glinidas
- [ ] Inhibidores de la alfaglucosidasa
- [ ] Aconsejo reducir dosis de (puede señalar más de una)
- [ ] Metformina
- [ ] iDPP4
- [ ] arGLP1
- [ ] iSCLT2
- [ ] Sulfonilureas
- [ ] Pioglitazona
- [ ] Glinidas
- [ ] Inhibidores de la alfaglucosidasa
- [ ] Aconsejo iniciar insulinización transitoria ante el posible riesgo de descompensación o necesidad de corticoides

15. **En un paciente con DM2 ya conocido que contrae COVID-19 y no está ingresado, ¿tiene en cuenta algún signo de alarma específico relacionado con la DM2 para indicar hospitalización?** Puede señalar más de una opción.

- [ ] Empeoramiento del control glucémico
- [ ] Aumento de la variabilidad glucémica
- [ ] Alteración en la determinación de cuerpos cetónicos
- [ ] Considero solamente los signos de alarma habituales en el seguimiento domiciliario del paciente positivo para SARS-CoV-2 (disnea, taquipnea, etc.)

BLOQUE 3. Hiperglucemia y COVID. Paciente hospitalizado

16. **En relación con la hiperglucemia al ingreso en pacientes hospitalizados por COVID-19, ¿en qué pacientes empeora el pronóstico?** Puede señalar más de una opción.

- [ ] Hiperglucemia en pacientes sin M2

- ☐ Hiperglucemia en pacientes con DM2
- ☐ En ninguno de ellos
- ☐ En ambos casos

17. **En su centro, si se determina la glucemia en el paciente sin diabetes conocida que ingresa por COVID-19, ¿cuál es la forma habitual?**
    - ☐ Se determina HbA1c sistemáticamente al ingreso
    - ☐ Se determina glucemia mediante analítica de laboratorio al ingreso
    - ☐ Se hace glucemia capilar sistemáticamente al ingreso
    - ☐ Se hace glucemia capilar diaria sistemáticamente
    - ☐ Se hace glucemia capilar sistemáticamente a los pacientes en tratamiento con glucocorticoides
    - ☐ No se determina de forma sistemática

18. **En su experiencia, ¿presenta un paciente con COVID-19 mayor riesgo de descompensación glucémica?**
    - ☐ Sí, siempre
    - ☐ Sí, pero solamente los pacientes con DM2 previa
    - ☐ Sí, pero solamente los pacientes con DM2 previa y mal controlados
    - ☐ Sí, pero solamente pacientes con comorbilidades previas g/o fragilidad
    - ☐ Sí, pero solo si recibe corticoides

19. **Para el abordaje del paciente sin DM2 conocida que es hospitalizado por COVID-19, presenta hiperglucemia y no precisa tratamiento con glucocorticoides**. Puede señalar más de una opción.
    - ☐ Solicito determinación de HbA1c si glucemia basal > 140 mg/dl y/o glucemia vespertina > 180 mg/dl
    - ☐ Si glucemia basal < 140 mg/dl realizo monitorización de la glucemia si está en tratamiento con corticoides
    - ☐ Si glucemia basal entre 140 y 180 mg/dl añado iDPP4 en el desayuno
    - ☐ Si glucemia basal > 180 mg/dl añado iDPP4 + insulina basal
    - ☐ Si glucemia basal > 180 mg/dl añado insulina basal + bolo
    - ☐ Realizo titulación de insulina basal cada 48 h y ajustes de unidades según controles
    - ☐ Manejo con protocolos propios de mi centro distintos a los enunciados
    - ☐ Mantengo su tratamiento habitual
    - ☐ Solicito siempre determinación de HbA1c
    - ☐ Solicito HbA1c si no tengo una de los 3 meses previos

BLOQUE 4. DM2 y COVID. Paciente hospitalizado con DM2 y COVID

20. **En el paciente con DM2 hospitalizado en planta por COVID-19, señale las medidas que considera adecuadas para reducir el riesgo de morbimortalidad**. Puede señalar más de una opción.
    - ☐ Controles de glucemia diarios
    - ☐ Indicación de sistemas de monitorización continua de la glucosa
    - ☐ Vigilar la variabilidad glucémica en los controles
    - ☐ Intensificar el tratamiento antidiabético si los controles están fuera de objetivos
    - ☐ Realizar cribado/seguimiento de signos o parámetros sugerentes de complicaciones (cetoacidosis, tormenta de citoquinas, complicaciones protrombóticas, hipoglucemia, hiperglucemia, hiperosmolaridad)
    - ☐ Realizar control del balance de líquidos
    - ☐ Ajuste de necesidades nutricionales
    - ☐ Pasar a manejo de la hiperglucemia con insulina (con o sin otros antidiabéticos)

21. **En el paciente con DM2 que ingresa por COVID-19, ¿con qué frecuencia se indica la determinación de glucemias capilares?** Puede señalar más de una opción.

- ☐ Ayunas/basal
- ☐ Antes de cada comida principal (desayuno, comida, cena)
- ☐ Antes de cada comida que haga el paciente (desayuno, comida, merienda y cena)
- ☐ Determinación preprandial y posprandial

22. **En su ámbito de trabajo, ¿tiene disponibilidad para prescribir sensores de monitorización continua de la glucosa en pacientes con DM2 que ingresan por COVID-19?** Puede señalar más de una opción.

- ☐ No
- ☐ Sí, según mi criterio
- ☐ Sí, pero solo en casos seleccionados (puede señalar más de una opción):
- ☐ Pacientes hospitalizados con mal control glucémico
- ☐ Pacientes hospitalizados con alta variabilidad glucémica
- ☐ Pacientes ambulatorios/hospitalizados con mal control glucémico
- ☐ Pacientes con comorbilidades o condicionantes
- ☐ Sospecha de hipoglucemias inadvertidas
- ☐ Situaciones puntuales intercurrentes (enfermedad aguda, épocas de alta actividad física, etc.)
- ☐ Pacientes que realizan actividad física intensa
- ☐ Educación terapéutica al inicio del diagnóstico

23. **Para el tratamiento de la hiperglucemia en el paciente con DM2 que es hospitalizado por COVID-19, ¿sigue habitualmente algún/algunos de estos criterios?** Puede señalar más de una opción.

- ☐ Marco como objetivo glucemia basal < 140 mg y glucemias resto del día entre 140 y 180 mg/dl
- ☐ En paciente con edad avanzada o frágiles contemplo objetivos más laxos (glucemia < 200 mg/dl)
- ☐ Si glucemia basal < 140 mg/dl y/o glucemia no basal entre 140–180 mg/dl, Manejo con iDPP4 + insulina basal (inicio 0,2 UI) + correcciones de insulina rápida si precisa
- ☐ Si glucemia basal entre 140 y 180 mg/dl y/o glucemia no basal > 180 mg/dl, manejo con iDPP4 + insulina basal (0,3–04 UI) + correcciones de insulina rápida si precisa
- ☐ Si glucemia basal > 180 mg/dl y/o glucemia no basal > 200 mg/dl, Manejo con insulina basal (0,3–0,4 UI) + correcciones de insulina rápida si precisa
- ☐ Si glucemias > 300 mg/dl mantenidas, inicio insulinización I.V.
- ☐ Realizo titulación de insulina basal cada 48 h y ajustes de unidades según controles
- ☐ Manejo con protocolos propios de mi centro distintos a los enunciados

24. **En el paciente con DM2 que es hospitalizado por COVID-19, numere en orden ascendente de mayor a menor relevancia los factores que tiene usted en cuenta para elegir el tratamiento hipoglucemiante.** Señale con un “1” el más relevante y así sucesivamente. Si alguno lo considera no relevante, indique un “0”.

- ☐ HbA1c de los últimos 3–6 meses
- ☐ HbA1c al ingreso
- ☐ Valor de glucemia al ingreso
- ☐ Tratamiento que tiene el paciente en domicilio
- ☐ Evitar las hipoglucemias
- ☐ Evitar la cetoacidosis
- ☐ Evitar las interacciones con otros fármacos que precise el paciente
- ☐ Evitar los posibles efectos negativos de los fármacos sobre la COVID-19
- ☐ Considerar las comorbilidades y condicionantes del paciente

25. **En el paciente con DM2 y COVID hospitalizado, ¿qué grupo terapéutico mantiene durante la hospitalización? Puede señalar más de una opción.**

- ☐ Metformina
- ☐ iDPP4
- ☐ arGLP1
- ☐ iSGLT2
- ☐ Sulfonilureas
- ☐ Pioglitazona
- ☐ Glinidas
- ☐ Inhibidores de la alfaglucosidasa

26. **En su experiencia, ¿qué porcentaje aproximado de pacientes hospitalizados con DM2 y COVID-19 precisan soporte nutricional artificial?**

- ☐ <10%
- ☐ 10–19%
- ☐ 20–29%
- ☐ 30–39%
- ☐ 40–49%
- ☐ >50%

27. **En el paciente con DM2 y COVID hospitalizado, si el paciente precisa soporte nutricional oral/enteral/parenteral, ¿emplea habitualmente fórmulas nutricionales específicas de diabetes?**

- ☐ Sí, tanto oral como enteral como parenteral
- ☐ Sí en oral g/o enteral, pero no en parenteral
- ☐ Lo tengo en cuenta en la nutrición parenteral, pero no en la oral/enteral
- ☐ No es algo prioritario para mí en caso de precisar soporte nutricional

28. **En el paciente que ha sido hospitalizado con DM2 y COVID-19, ¿qué actitud suele tomar con mayor frecuencia en relación con el tratamiento hipoglucemiante al alta? Puede señalar más de una opción.**

- ☐ Habitualmente vuelvo al tratamiento que tenía el paciente antes del ingreso
- ☐ Mantengo el tratamiento del hospital, con ajuste de dosis
- ☐ Indico metformina + iDDP4 y ajuste de intensificación por el médico de familia si precisa
- ☐ Otros

BLOQUE 5. Hiperglucemia de estrés por COVID/hiperglucemia por esteroides

29. **Para el tratamiento de la hiperglucemia en el paciente con diabetes esteroidea sin diabetes previa conocida en el ámbito hospitalario, ¿sigue habitualmente algún/algunos de estos criterios?** Puede señalar más de una opción.

- ☐ Si añado esteroides, realizo controles de glucemia durante los siguientes días
- ☐ Marco como objetivo glucemia basal (GB) < 140 mg y glucemias posprandiales (GPP) < 200 mg/dl
- ☐ Inicio tratamiento con iDPP4
- ☐ Si no se alcanzan objetivos con iDPP4 y GB > 140 mg/dl + GPP > 200 mg/dl, añado insulina NPH/NPL
- ☐ Si no se alcanzan objetivos con iDPP4 y GB >140 mg/dl + GPP > 200 mg/dl, añado insulina prandial (con desayuno y almuerzo)
- ☐ Si no se alcanzan objetivos con iDPP4 y GB > 140 mg/dl + GPP > 200 mg/ dl, añado insulina glargina/detemir
- ☐ Si no se alcanzan objetivos con iDPP4 y GB >140 mg/dl + GPP > 200 mg/ dl, añado insulina prandial (con ajustes según controles)
- ☐ Si glucemias > 300 mg/dl mantenidas, inicio insulinización i.v.

- [ ] Manejo con protocolos propios de mi centro distintos a los enunciados

30. **Para el tratamiento de la hiperglucemia secundaria a esteroides en el paciente con DM2 previa, ¿sigue habitualmente algún/algunos de estos criterios?** Puede señalar más de una opción.
    - [ ] Si añado esteroides, realizo controles de glucemia durante los siguientes días
    - [ ] Marco como objetivo glucemia basal (GB) < 140 mg y glucemias posprandiales (GPP) < 200 mg/dl
    - [ ] Inicio tratamiento con iDPP4
    - [ ] Si glucemia basal < 140 mg/dl y/o glucemia no basal > 180 mg/dl, Manejo con iDPP4 + insulina basal (inicio 0,2 UI) + correcciones de insulina rápida si precisa
    - [ ] Si glucemia basal entre 140 y 180 mg/dl, manejo con iDPP4 + insulina basal (0,3–0,4 UI) + correcciones de insulina rápida si precisa
    - [ ] Si glucemia basal > 180 mg/dl g/o glucemia no basal > 200 mg/dl, Manejo con insulina basal (0,3–0,4 UI) + correcciones de insulina rápida si precisa
    - [ ] Si glucemias > 300 mg/dl mantenidas, inicio insulinización I.V.
    - [ ] Realizo titulación de insulina basal cada 48 h y ajustes de unidades según controles
    - [ ] Si no se controla con insulina basal, planteo tratamiento con insulina NPH/ NPL o glargina/detemir según cifras de GB y GGP
    - [ ] Manejo con protocolos propios de mi centro distintos a los enunciados

31. **Piense en el paciente con DM2 ga conocido que es hospitalizado por COVID-19 y precisa tratamiento con glucocorticoides. ¿Qué grupo terapéutico mantiene durante la hospitalización?** Puede señalar más de una opción.
    - [ ] Metformina
    - [ ] iDPP4
    - [ ] arGLP1
    - [ ] iSCLT2
    - [ ] Sulfonilureas
    - [ ] Pioglitazona
    - [ ] Glinidas
    - [ ] Inhibidores de la alfaglucosidasa

BLOQUE 6. Algoritmos y recursos

32. **La SEMI ha planteado un algoritmo para el abordaje de la hiperglucemia hospitalaria en la COVID-19. ¿Qué opina?**
    - [ ] No lo conocía
    - [ ] Lo conozco, pero no estoy de acuerdo
    - [ ] Lo conozco y estoy de acuerdo parcialmente
    - [ ] Lo conozco y estoy de acuerdo

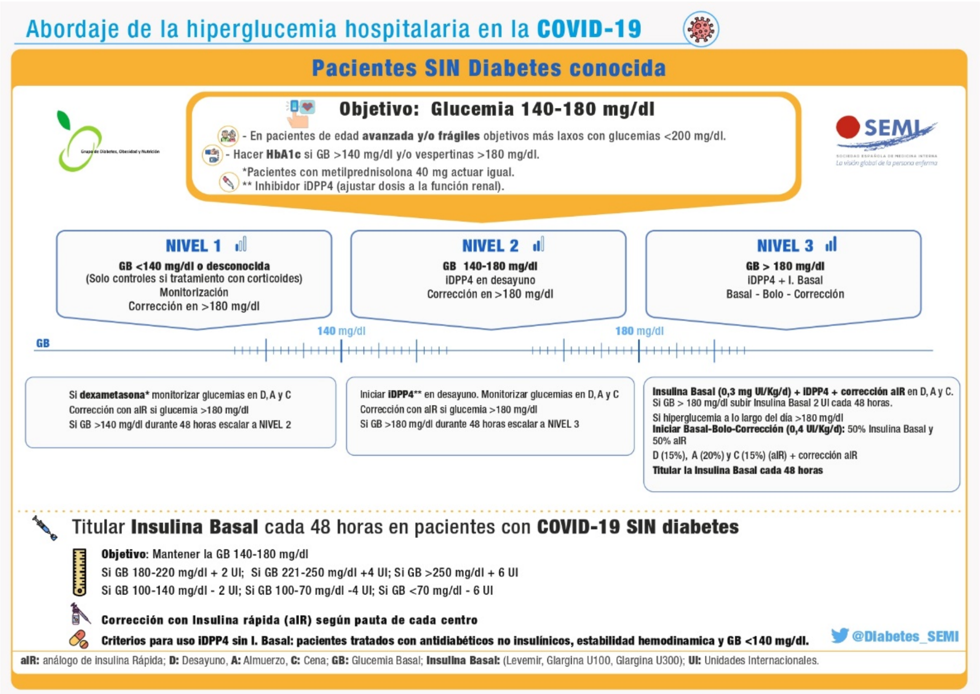

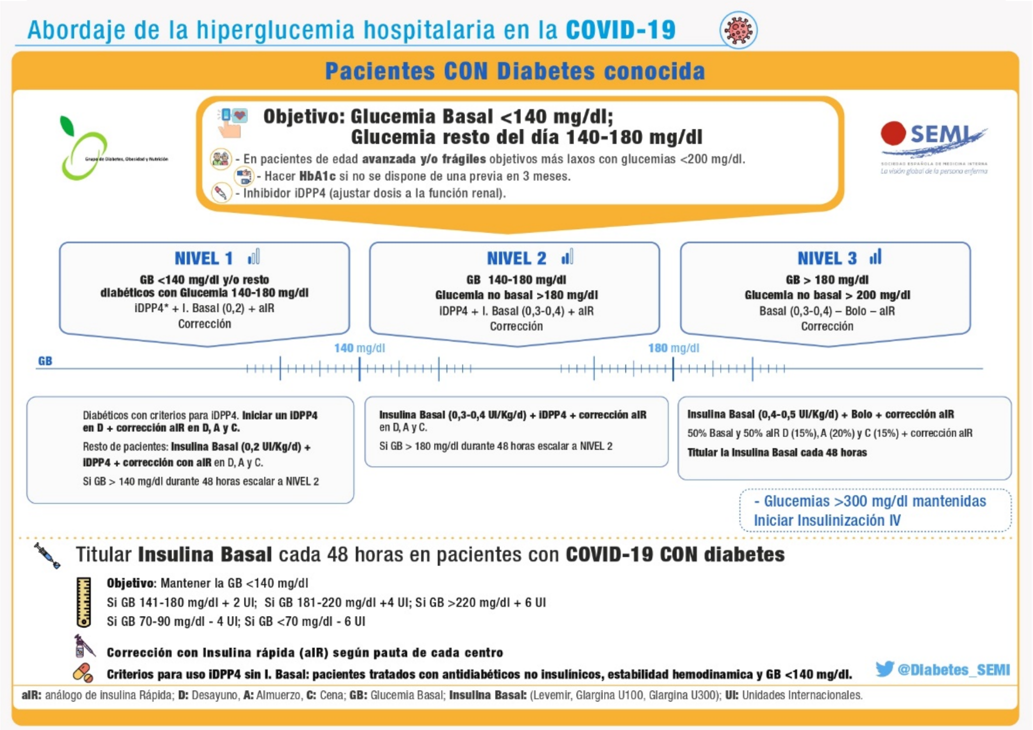

33. **La SEMI ha planteado un algoritmo para el abordaje de la diabetes esteroidea o hiperglucemia secundaria a esteroides en el contexto de la pandemia de la COVID-19. ¿Qué opina?**

- ☐ No lo conocía
- ☐ Lo conozco, pero no estoy de acuerdo
- ☐ Lo conozco y estoy de acuerdo parcialmente
- ☐ Lo conozco y estoy de acuerdo

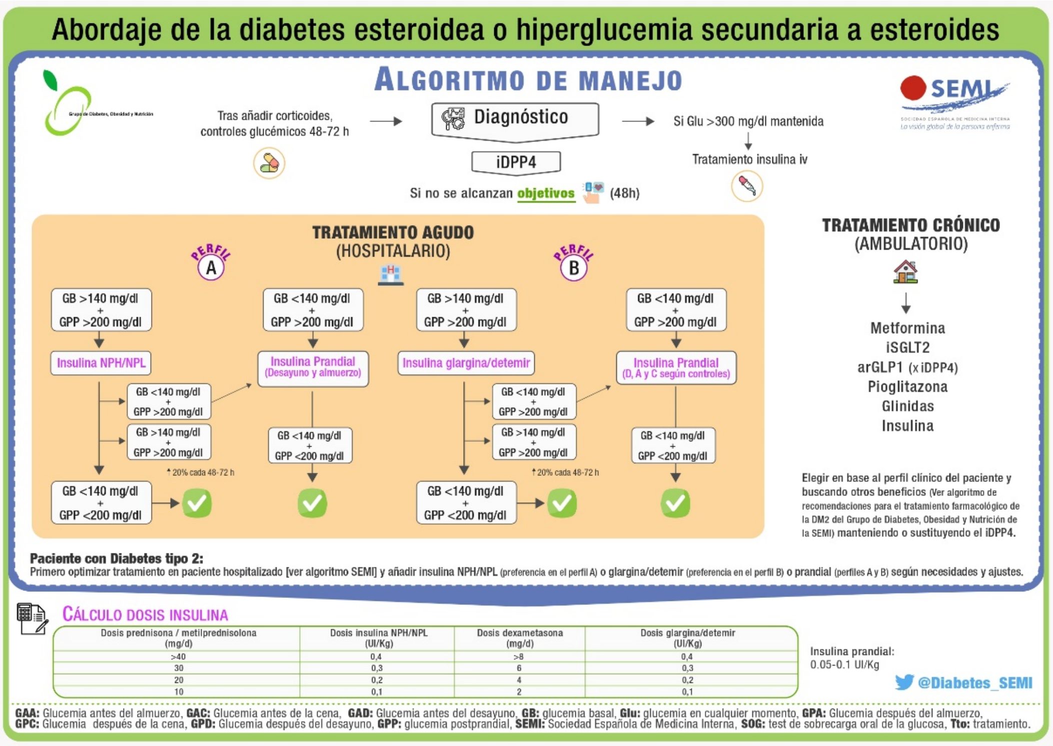
Abordaje de la diabetes esteroidea o hiperglucemia secundaria a esteroides
Grupo de Diabetes, Obesidad y Nutrición
ALGORITMO DE MANEJO
SEMI
SOCIEDAD ESPAÑOLA DE MEDICINA INTERNA
La visión global de la persona enferma
Tras añadir corticoides, controles glucémicos 48-72 h
Diagnóstico
iDPP4
Si Glu >300 mg/dl mantenida
Tratamiento insulina iv
Si no se alcanzan objetivos (48h)
TRATAMIENTO AGUDO (HOSPITALARIO)
PERFIL A
PERFIL B
GB >140 mg/dl + GPP >200 mg/dl
Insulina NPH/NPL
GB <140 mg/dl + GPP >200 mg/dl
GB >140 mg/dl + GPP >200 mg/dl
↑20% cada 48-72 h
GB <140 mg/dl + GPP <200 mg/dl
GB <140 mg/dl + GPP >200 mg/dl
Insulina Prandial (Desayuno y almuerzo)
GB <140 mg/dl + GPP <200 mg/dl
GB >140 mg/dl + GPP >200 mg/dl
Insulina glargina/detemir
GB <140 mg/dl + GPP >200 mg/dl
GB >140 mg/dl + GPP >200 mg/dl
↑20% cada 48-72 h
GB <140 mg/dl + GPP <200 mg/dl
GB <140 mg/dl + GPP >200 mg/dl
Insulina Prandial (D, A y C según controles)
GB <140 mg/dl + GPP <200 mg/dl
TRATAMIENTO CRÓNICO (AMBULATORIO)
Metformina
iSGLT2
arGLP1 (x iDPP4)
Pioglitazona
Glinidas
Insulina
Elegir en base al perfil clínico del paciente y buscando otros beneficios (Ver algoritmo de recomendaciones para el tratamiento farmacológico de la DM2 del Grupo de Diabetes, Obesidad y Nutrición de la SEMI) manteniendo o sustituyendo el iDPP4.
Paciente con Diabetes tipo 2:
Primero optimizar tratamiento en paciente hospitalizado [ver algoritmo SEMI] y añadir insulina NPH/NPL (preferencia en el perfil A) o glargina/detemir (preferencia en el perfil B) o prandial (perfiles A y B) según necesidades y ajustes.
CÁLCULO DOSIS INSULINA
Dosis prednisona / metilprednisolona (mg/d) | Dosis insulina NPH/NPL (UI/Kg) | Dosis dexametasona (mg/d) | Dosis glargina/detemir (UI/Kg)
>40 | 0,4 | >8 | 0,4
30 | 0,3 | 6 | 0,3
20 | 0,2 | 4 | 0,2
10 | 0,1 | 2 | 0,1
Insulina prandial: 0.05-0.1 UI/Kg
@Diabetes_SEMI
GAA: Glucemia antes del almuerzo, GAC: Glucemia antes de la cena, GAD: Glucemia antes del desayuno, GB: glucemia basal, Glu: glucemia en cualquier momento, GPA: Glucemia después del almuerzo, GPC: Glucemia después de la cena, GPD: Glucemia después del desayuno, GPP: glucemia postprandial, SEMI: Sociedad Española de Medicina Interna, SOG: test de sobrecarga oral de la glucosa, Tto: tratamiento.

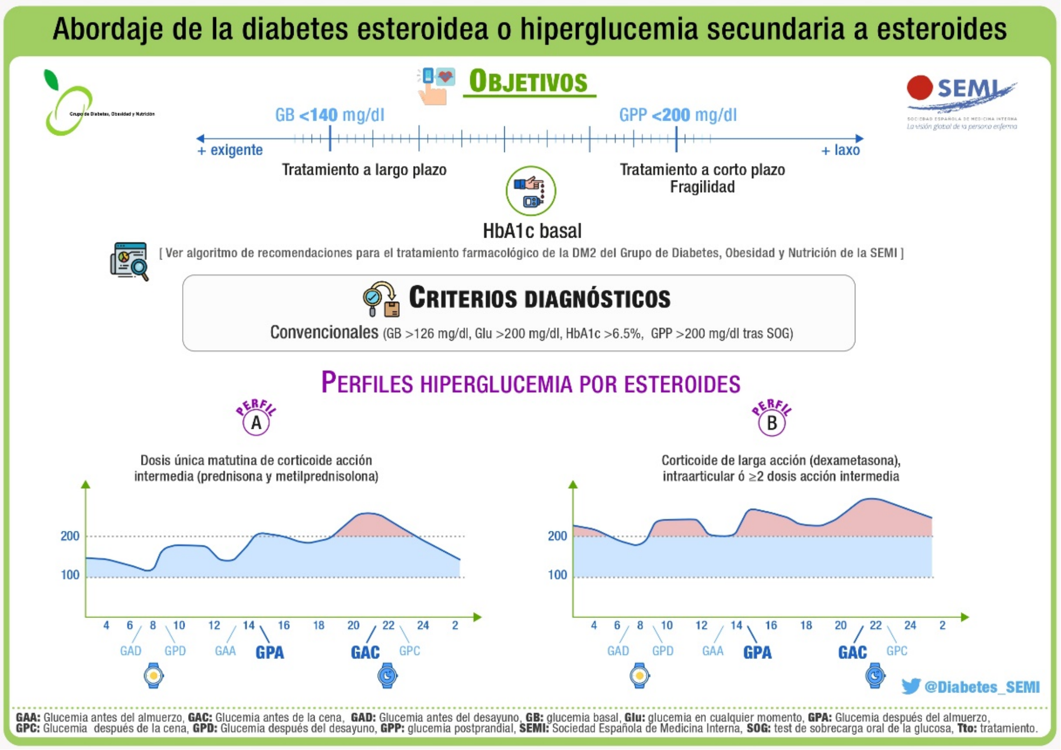
Abordaje de la diabetes esteroidea o hiperglucemia secundaria a esteroides
Grupo de Diabetes, Obesidad y Nutrición
OBJETIVOS
SEMI
SOCIEDAD ESPAÑOLA DE MEDICINA INTERNA
La visión global de la persona enferma
GB <140 mg/dl
GPP <200 mg/dl
+ exigente
+ laxo
Tratamiento a largo plazo
Tratamiento a corto plazo
Fragilidad
HbA1c basal
[Ver algoritmo de recomendaciones para el tratamiento farmacológico de la DM2 del Grupo de Diabetes, Obesidad y Nutrición de la SEMI]
CRITERIOS DIAGNÓSTICOS
Convencionales (GB >126 mg/dl, Glu >200 mg/dl, HbA1c >6.5%, GPP >200 mg/dl tras SOG)
PERFILES HIPERGLUCEMIA POR ESTEROIDES
PERFIL A
Dosis única matutina de corticoide acción intermedia (prednisona y metilprednisolona)
200
100
4 6 8 10 12 14 16 18 20 22 24 2
GAD GPD GAA GPA GAC GPC
PERFIL B
Corticoide de larga acción (dexametasona), intraarticular ó ≥2 dosis acción intermedia
200
100
4 6 8 10 12 14 16 18 20 22 24 2
GAD GPD GAA GPA GAC GPC
@Diabetes_SEMI
GAA: Glucemia antes del almuerzo, GAC: Glucemia antes de la cena, GAD: Glucemia antes del desayuno, GB: glucemia basal, Glu: glucemia en cualquier momento, GPA: Glucemia después del almuerzo, GPC: Glucemia después de la cena, GPD: Glucemia después del desayuno, GPP: glucemia postprandial, SEMI: Sociedad Española de Medicina Interna, SOG: test de sobrecarga oral de la glucosa, Tto: tratamiento.
